# Editorial: Genomic selection and evolution in domestic animals

**DOI:** 10.3389/fvets.2026.1904348

**Published:** 2026-07-01

**Authors:** Xuexue Liu, Hojjat Asadollahpour Nanaei

**Affiliations:** Centre d'Anthropobiologie et de Génomique de Toulouse, Centre national de la recherche scientifique (CNRS), University of Toulouse, Toulouse, France

**Keywords:** animal breeding and genetics, domestication, evolutionary genomics, genomic technologies, livestock productivity

## Introduction

Over the past two decades, advances in genomics have dramatically reshaped research and breeding strategies in domestic animals. Advances in high-throughput genotyping, sequencing technologies, and computational genomics have greatly expanded researchers' ability to investigate the genetic architecture of complex traits, reconstruct evolutionary histories, and design more effective breeding programs. This Research Topic, “*Genomic Selection and Evolution in Domestic Animals*,” brings together recent studies at the intersection of quantitative genetics, molecular genomics, and animal breeding, across a range of livestock species including cattle, sheep, goats, horses, and yaks. The 15 original research articles included in this Research Topic highlight the diversity and rapid development of contemporary animal genomics research. Together, they explore how genetic variation influences phenotypic traits, how domestic animal populations adapt to different environmental conditions, and how genomic information can be applied to improve breeding efficiency and productivity. Collectively, these contributions advance our understanding of both the evolutionary forces that have shaped domestic animal genomes and the practical tools needed to exploit genomic data in breeding and production contexts.

## Genomic prediction and selection strategies

Accurate estimation of genomic breeding values remains a major challenge in modern animal breeding, particularly when pedigree information or genotyping data are incomplete or unreliable. Rocha et al. address this directly by evaluating genotyping strategies for single-step genomic predictions in sheep under various pedigree error scenarios. Their simulations showed that both the type and frequency of pedigree errors can significantly influence prediction accuracy. The study also provides practical insight into cost-effective genotyping approaches capable of maintaining reliable predictions under less than ideal data conditions, which is especially relevant for commercial sheep breeding programs. For dairy cattle, feed efficiency is an economically and environmentally critical trait. Chegini et al. compared different modeling approaches for predicting genomic breeding values related to feed efficiency in lactating dairy cows. Their findings showed that the choice of model, including residual feed intake, efficiency ratios, and related measures can substantially affect both the accuracy and bias of genomic predictions. Their results highlight the need to carefully consider trait definition when implementing genomic selection for feed efficiency, with implications for sustainable livestock production.

The development of efficient genotyping platforms is essential for the wider adoption of cost-effective genomic selection programs. Guo et al. described the design, validation, and application of a 1K liquid chip for genome-wide association analysis in Alpine Merino sheep. This low-density chip, validated against denser panels, offers a practical and affordable platform for population-scale genotyping, facilitating the implementation of genomic selection in resource-limited breeding contexts.

## Population genetics, inbreeding, and genetic diversity

Maintaining genetic diversity while achieving genetic gain is a longstanding tension in animal breeding. Cartuche-Macas et al. examined reproductive performance, inbreeding evolution, and genetic diversity in the Venezuelan Carora cattle breed through pedigree analysis, revealing trends in inbreeding accumulation and effective population size that raise concerns for long-term breed viability. Their work underscores the importance of systematic pedigree monitoring in locally adapted breeds that may have limited global recognition.

At the genomic level, runs of homozygosity (ROH) have become an important tool for studying inbreeding and selection history. Qi et al. conducted a genome-wide assessment of ROH and inbreeding in Inner Mongolia cashmere goats, and identified several candidate genes located within ROH islands that are associated with economically important traits, such as fiber quality and body morphology. Their findings not only shed light on the genetic consequences of selection in this breed but also pinpoint targets for future genomic improvement programs.

## Trait architecture: GWAS and candidate gene discovery

Genome-wide association studies (GWAS) remain a key approach in livestock genomics for uncovering the genetic basis of both complex and Mendelian traits. In this Research Topic two complementary studies examined mandibular defects in Duolang sheep, a welfare-relevant condition with a strong genetic component. Fang et al. applied GWAS to identify key genes associated with mandibular prognathism in Duolang sheep, while Cao et al. used genome-wide selection analysis to identify genes linked to mandibular defects in the same breed. Although the two studies used different analytical strategies, they point toward overlapping candidate regions that merit further functional validation and may ultimately support marker-assisted selection to reduce the incidence of this defect. The genetic origin and phenotypic effects of the double-muscle mutation in the myostatin gene (*MSTN*) were investigated by Xu, Gao, et al. in a Hu × East Friesian hybrid sheep population. Their analysis traces the mutational history of this functionally important variant and quantifies its effects on growth traits, providing insights into both the evolutionary dynamics of this mutation in sheep and its potential utility in commercial meat production.

Molecular genetic tools are also being refined for practical diagnostics and population monitoring. Xu, Yang, et al. developed a rapid visual detection method for indel polymorphisms in the bovine PRNP gene using a duplex MIRA-LFD assay. This portable and sensitive approach enables field-level genotyping for prion disease susceptibility alleles, with implications for disease control programs and breeding decisions in cattle.

## Adaptation, multi-omics, and functional genomics

Several contributions to this Research Topic investigate how domestic animals respond to challenging environments at the molecular level, particularly at high altitude. Akhmiyati et al. employed a multi-omics approach to reveal muscle quality divergence between high-altitude Bayinbuluke and low-altitude Turpan black sheep, identifying differences in metabolite profiles, gene expression, and protein composition that underlie phenotypic adaptation. Chen et al. extended this framework to the digestive system, using integrated metabolomic and transcriptomic analysis to reveal digestive tract adaptations to high altitude in Bayanbulak sheep. Together, these studies highlight the multi-layered molecular remodeling that accompanies high-altitude adaptation in ruminants.

Complementary insights into exercise-induced molecular responses come from the equine context. Ma et al. analyzed miRNA expression in Yili horses before and after a 5,000-meter race, identifying differentially expressed miRNAs linked to muscle function, energy metabolism, and stress response pathways. This study represents a valuable contribution to equine exercise genomics and may have relevance for performance horse selection and welfare monitoring.

The molecular regulation of milk production traits was explored in two separate studies. Zhao et al. investigated the effects of FGF2 on ovine mammary epithelial cells and milk production traits in dairy sheep, demonstrating that this growth factor modulates cell proliferation, differentiation, and milk synthesis—findings with potential applications in improving dairy sheep productivity. In yaks, Zheng et al. investigated the effects of TRIB3 gene overexpression and interference on lipid metabolism in mammary epithelial cells, identifying a regulatory role for TRIB3 in milk fat synthesis that could be leveraged in yak breeding programs targeting milk composition.

Adipose-derived stem cells (ADSCs) from sheep tails provide a useful model for studying fat deposition, a trait of major economic importance in many sheep breeds. Han et al. showed that PDGFD helps maintain ovine tail ADSCs in a proliferative state by suppressing CXCL8 and activating PI3K/MAPK signaling, revealing a molecular pathway involved in regulating adipogenesis in this tissue. These findings improve our understanding of fat tail development and may guide future efforts to modulate this trait through genomic or nutritional interventions.

## Conclusion

The articles assembled in this Research Topic collectively illustrate the remarkable progress being made at the interface of genomics, evolutionary biology, and animal production science. From improved methods for genomic prediction and chip design, to multi-omics dissection of environmental adaptation, to GWAS-based identification of trait loci, these contributions demonstrate the power of genomic approaches to transform our understanding of domestic animals. As sequencing costs continue to decline and computational methods continue to improve, the integration of genomic, transcriptomic, proteomic, and metabolomic data is likely to become more common in both research and applied breeding contexts.

We are grateful to all contributing authors for their high-quality work and to the reviewers for their rigorous and constructive evaluations. We hope that this Research Topic will serve as a useful resource for researchers, breeders, and policymakers working to advance more sustainable and productive domestic animal systems.

